# Endogenous fMRI default mode network fluctuations both positively and negatively correlate with individual transfer of learning

**DOI:** 10.3389/fnsys.2014.00229

**Published:** 2014-12-01

**Authors:** Raphael Kaplan

**Affiliations:** ^1^Department of Information and Communication Technologies, Center for Brain and Cognition, Computational Neuroscience Group, Universitat Pompeu FabraBarcelona, Spain; ^2^Wellcome Trust Centre for Neuroimaging, University College LondonLondon, UK

**Keywords:** intrinsic, fMRI, learning, hippocampus, vmPFC, memory

The behavioral relevance of resting-state/endogenous default mode network (DMN) fluctuations, particularly in the medial temporal lobe (MTL), is a question of great interest to cognitive neuroscientists. Most studies relating MTL endogenous fluctuations with behavior have focused on the hippocampus, which despite its sometimes-inconsistent coupling (Huijbers et al., 2011; Ward et al., 2014), is traditionally linked with the DMN (Buckner et al., 2008). Several approaches have related endogenous hippocampal fMRI signal fluctuations to behavior. Past approaches have included comparing trait-level amplitude changes in task vs. rest with memory performance (Wig et al., 2008), comparing endogenous functional connectivity before and after learning with memory performance (Tambini et al., 2010), and correlating endogenous functional connectivity prior to a memory task with task performance (Wang et al., 2010).

Imagination and fictive planning, behaviors that implicitly recapitulate previously learned information into novel representations, are thought to utilize DMN regions such as the MTL, medial prefrontal cortex (mPFC), and precuneus/posterior cingulate cortex (PCC; Buckner et al., 2008). Transfer of learning, using previously learned information in order to make novel inferences, notably recruits similar brain regions (Zeithamova et al., 2012). Transfer of learning is highly variable among subjects (Wimmer and Shohamy, 2012) and theoretically there should be trait markers of an individual's ability observable in endogenous fluctuations. A recent study by Gerraty et al. (2014), investigated potential trait markers of transfer of learning by combining behavioral testing with measurement of endogenous fMRI fluctuations in areas like the ventromedial PFC (vmPFC) and hippocampus within the same subjects.

In the transfer of learning task, Gerraty et al. (2014) had subjects repeatedly view four pairs of upright neutral faces during performance of an unrelated cover task. The authors referred to one face from each pair as an S_1_ face and the other as a S_2_ face. Afterwards, subjects then learned which S_2_ faces from each viewed pair could lead to monetary loss or gain. Finally, subjects were then instructed to choose the more rewarding of one of two faces. Subjects chose both between S_1_ faces, the faces that had no reward association, and also between S_2_ faces, the faces that sometimes had a previous reward association. The authors hypothesized that choice bias for choosing the more rewarding S_2_ face would carry over, i.e., transfer of learning, to the S_1_ faces that the S_2_ faces were originally associated with. Approximately several days after subjects' task participation, they underwent two separate 5-min fMRI measurements of endogenous functional connectivity.

The authors defined their regions of interest from prominent networks observed using a statistical technique that uncovers hidden components contributing to a signal, independent component analysis (ICA). They specifically focused on analyzing endogenous functional connectivity in two regions associated with transfer of learning, the vmPFC and hippocampus. The authors also investigated endogenous functional connectivity in the DMN and two other networks. Subjects' performance on the reward learning, choosing the S_2_ face that was more rewarding, was close to ceiling. Subjects' transfer of learning performance, i.e., choosing S_1_ faces originally paired with rewarding S_2_ faces over S_1_ faces originally paired with less rewarding S_2_ faces, was significantly lower, but still above chance.

Using ICA, the authors identified a ventral medial network (VMN) of correlated activity that partially overlapped anatomically with the DMN, that included the PCC, frontal pole, and a vmPFC cluster extending into the bilateral ventral striatum and bilateral anterior hippocampus. The peak voxels in the five different brain regions located within the vmPFC cluster, consisting of peaks in the vmPFC and bilateral anterior hippocampus and ventral striatum, were then correlated with each other. Left anterior hippocampal endogenous functional connectivity with the vmPFC negatively correlated with individual transfer of learning performance (Gerraty et al., 2014). The authors then examined whether endogenous activity in either region correlated with the functional connectivity of the DMN. Separately, vmPFC and hippocampal functional connectivity with the rest of the DMN both correlated with transfer of learning performance, but the correlations were in opposite directions. Functional connectivity between vmPFC-DMN positively predicted transfer of learning performance, while hippocampal-DMN functional connectivity was negatively correlated. This result suggests that fMRI signals within different regions of an endogenous network like the DMN can correlate with the same behavior in different ways.

An important question remains unanswered by this study, what does the strength of fMRI endogenous correlations between brain regions and the direction of the correlation mean for individual performance? Does increased correlation strength between brain regions mean increased communication between regions? Notably, the authors find that decreased endogenous functional connectivity between the task-relevant regions and the left hippocampus correlates with better individual transfer of learning performance, while many of the studies looking at hippocampal endogenous fluctuations find the opposite (albeit with different tasks and functional connectivity with different brain regions), that increased amplitude or interregional correlation strength predicts enhanced performance (Wig et al., 2008; Tambini et al., 2010; Wang et al., 2010). Furthermore, the authors speculate that heightened vmPFC-hippocampus endogenous fMRI functional connectivity might be found in schizophrenia patients compared to healthy controls, yet reduced mPFC-hippocampal endogenous functional connectivity has already been reported in schizophrenic patients compared to healthy controls (Zhou et al., 2008). This discrepancy might be due to the difficulty of determining which metric among functional connectivity, regional amplitude changes, or graph theoretical measures, most closely parallels fMRI activity induced during explicit experimental tasks. Future studies could better clarify how correlated endogenous fMRI fluctuations in different brain regions relate to neural communication and consequently behavior, by incorporating work such as He and colleagues' (2008) paper linking slow cortical potentials with endogenous fMRI networks into their hypotheses.

The paper's key accomplishment is relating commonly observed endogenous fMRI DMN fluctuations in the vmPFC and hippocampus to individual behavioral performance. Another implication is that resting-state functional connectivity might serve as a trait marker of individual learning ability that could be modified with improved performance through behavioral training, or potentially disrupted by disease pathology. Still, as Gerraty et al., stress, the link between individual differences found in fMRI signal changes during task paradigms and endogenous functional connectivity is unclear and needs replicable, theoretically validated metrics to create a stronger relationship between endogenous fluctuations and behavior.

## Conflict of interest statement

The author declares that the research was conducted in the absence of any commercial or financial relationships that could be construed as a potential conflict of interest.
